# When Do Coworkers’ Idiosyncratic Deals Trigger Social Undermining?—The Moderating Roles of Core Self-Evaluations and Conscientiousness

**DOI:** 10.3389/fpsyg.2022.866423

**Published:** 2022-05-25

**Authors:** Jingwen Wang, Jun Ma

**Affiliations:** School of Management, Shanghai University, Shanghai, China

**Keywords:** idiosyncratic deals, relative deprivation, social undermining, core self-evaluations, conscientiousness

## Abstract

Idiosyncratic deals are personalized work arrangements negotiated between enterprises and employees based on employees’ abilities and needs, previous studies have focused more on their positive effects on i-dealers and neglected the negative effects on peers in the process of interpersonal interaction. In view of this, this study explores the effects of coworkers’ idiosyncratic deals on employees’ social undermining and the internal mechanism based on social comparison theory. This study tested the theoretical model with a sample of 331 employees from six enterprises in China. The results showed that the interaction between perceptions of coworkers’ receiving idiosyncratic deals and low core self-evaluations stimulated employees’ feelings of relative deprivation, which triggered social undermining toward i-dealers. At the same time, employees’ conscientiousness could weaken the positive effect of relative deprivation on social undermining. Therefore, it reveals the negative peer effect of idiosyncratic deals and provides theoretical and practical implications for preventing the interpersonal harm doing caused by idiosyncratic deals.

## Introduction

Corporate practices show that employee contributions are increasingly power-law distributed rather than the traditional normal distribution (Aguinis et al., 2012), meaning that organizational competitiveness depends on the value created by a small number of employees. To better motivate and retain these core employees, HRM practices are increasingly transitioning to a more idiosyncratically approach (Liao et al., 2016). A typical example is idiosyncratic deals proposed by Rousseau (2001). As an idiosyncratic working arrangement negotiated between employees and organizations that benefit both parties, including more flexible working hours, more opportunities for career development, and higher pay incentives, it has gained considerable attention as an HR strategy to improve employees’ loyalty and performance.

Studies have found that idiosyncratic deals have a significant positive impact on the recipients. They can increase recipients’ positive emotions (Van der Heijden et al., 2021), job performance (Maltarich et al., 2017; Kong et al., 2020), helping behavior (Guerrero and Challiol-Jeanblanc, 2016), and organizational citizenship behavior (Anand et al., 2018). Existing studies mostly focus on the positive effects on idiosyncratic deals recipients, only a few studies have paid attention to its negative effects on non-recipients. For example, it has been found that idiosyncratic deals can reduce peer motivation (Giarratana et al., 2018), deviant behaviors (Kong et al., 2020), and performance (Abdulsalam et al., 2021). However, these studies mostly started from an inter-individual perspective. The negative effects of idiosyncratic deals during the interpersonal interaction process on non-recipients still need to be further explored. Thus, to further understand the mechanisms in idiosyncratic deals on interpersonal relationships for improving the incentive effect, a deeper investigation is necessary.

Indeed, as a typical differentiation within an organization, idiosyncratic deals are reflected in the fact that recipients have more development opportunities, more resources, or more flexible work arrangements than non-recipients. Individuals are often accompanied by self-serving biases in the attribution process. When employees perceive that only core employees achieve idiosyncratic deals, they will change their perceptions (Garg and Fulmer, 2017) and feel the psychological gap. The horizontal comparison will make them think that the organization gives them fewer resources than i-deals, which may create a sense of relative deprivation. To eliminate the negative psychological experience of self-threatening and improve self-evaluations (Yu and Duffy, 2016), peers will take measures to vent their grievances and change the state (He et al., 2019). When they consider they have little potential for self-improvement through effort, they are likely to reduce coworkers’ achievements through harm doing behaviors. Social undermining behavior is an aggressive tool hindering the success of others and preventing others from establishing positive interpersonal relationships, which can fill the psychological gap of the perpetrator. Specially, social undermining is a covert and safe harmful behavior. The negative effects on others will not be immediately apparent, on the one hand, this can reduce the perpetrator’s guilt; on the other hand, it is also a safe means, not easily detected by others.

Research has shown that in the process of social comparison, people with different personality characteristics respond in different ways to the same comparison target and have different effects on the individual (Seidlitz et al., 1997), so exploring their important roles in the process of social comparison is an inevitable issue (Yu et al., 2018). Further, according to social comparison theory, we argue that the effects of perceptions of coworkers’ receiving i-deals on social undermining may vary depending on peers’ personality characteristics (core self-evaluations and conscientiousness). First, we tested the moderating effect of core self-evaluations on the relationship between perceptions of coworkers’ receiving i-deals and relative deprivation. Cohen-Charash (2009) stated that individuals’ reactions to social comparisons depend on their evaluations of themselves. Individuals with low core self-evaluations tend to believe they cannot achieve similar performance as i-dealers in the future and experience a sense of relative deprivation. This provides a theoretical basis for us to find the boundary condition of the effect between perceptions of coworkers’ receiving i-deals and relative deprivation. Second, the level of employees’ conscientiousness determines their achievement orientation and the attitude of responsibility for work (Costa and McCrae, 1992). It affects the way he or she works (Borghuis et al., 2017). It has been indicated that those high in conscientiousness tend to be achievement-oriented, able to work firmly toward their goals (Mount and Barrick, 1998), and follow ethical principles (McFerran et al., 2010). This can influence the effect of relative deprivation on social undermining in both cognitive and emotional aspects. Thus, this study chose conscientiousness as a moderator in the model and proposed that high level of conscientiousness may weaken the relationship between relative deprivation and social undermining.

In summary, based on social comparison theory, this paper introduces relative deprivation as a mediating variable, core self-evaluations, and conscientiousness as moderating variables, and constructs a two-stage mediating moderated model, aiming to investigate the mechanisms of the negative effects of coworkers’ idiosyncratic deals and how to mitigate such negative effects in order to help organizations better leverage idiosyncratic deals.

## Theory and Hypotheses

### Perceptions of Coworkers’ Receiving I-Deals, Core Self-Evaluations, and Relative Deprivation

Organizations are becoming more and more dependent on talents with specialized skills, and competition among organizations is increasingly becoming a competition for talents. In order to attract employees, organizations have to meet the special requirements of employees in certain aspects. Therefore, idiosyncratic deals have become an important motivational strategy for organizations to attract and retain core employees (Rousseau et al., 2006), and employees who have been given idiosyncratic deals have more initiative at work compared to other employees in the organization, which leads to variability in the compensation benefits received by different employees. This often stimulates a sense of inequity among peers and brings about negative peer effects (Abdulsalam et al., 2021).

Relative deprivation is defined as an individual’s or a group’s perception of their own inferiority in comparison with a given standard, which results in an angry or resentful emotional response (Smith et al., 2012). Relative deprivation occurs when individuals compare what they have been given with what others have received and feel that they have less than they deserve (Smith et al., 2012), with subsequent negative emotions and cognitions (Khan et al., 2013). In summary, the sense of relative deprivation is a response brought about by upward comparisons made by individuals in unfavorable status (Wood, 1989), which assesses the extent to which individuals perceive themselves to be in unfavorable status.

Idiosyncratic deals as a unique motivational approach, only a very small number of core employees in the organization can achieve. When employees have a high level of perceptions of coworkers’ receiving i-deals, it means that employees consider that their colleagues have gained more trust, attention, and respect in their interactions with leaders (Guerrero et al., 2016), have a higher status, and enjoy more opportunities and benefits (Ng and Lucianetti, 2016a). However, perceptions of coworkers’ receiving i-deals do not necessarily lead to negative effects (Garg and Fulmer, 2017), there are specific boundary conditions.

It is noteworthy that personality characteristics play an important role in the process of social comparison (Seidlitz et al., 1997). Therefore, exploring their role in the process of social comparison is an inevitable issue (Yu et al., 2018). It has been found that how an individual reacts to unfavorable social comparisons is influenced by perceived controllability, i.e., the individual’s perceived ability to make a difference (Cohen-Charash, 2009). Core self-evaluations consist of four basic characteristics: self-esteem, generalized self-efficacy, neuroticism (emotional stability), and locus of control (Judge et al., 1998), and is the most basic evaluation of an individual’s abilities and values (Zhao and Shi, 2018). Research has shown that employee core self-evaluation acts as a moderating variable that affects employees’ reactions and attitudes toward certain behaviors (Chang et al., 2012). Individuals with low core self-evaluation perceive themselves as less capable of solving problems and controlling things, and they need to rely on external information to regulate their motivation and behavior (Kluemper et al., 2018). Employees with low core self-evaluation feel powerless in the face of stress and threats (Judge et al., 1998) and often have a sense of being out of control when faced with challenges in their lives (Aryee et al., 2017). In comparison with i-deals, on the one hand, individuals with low core self-evaluation are more sensitive to negative information (Chang et al., 2012), feel pressured and threatened when faced with core employees gaining idiosyncratic deals they do not have, creating a sense of relative deprivation and taking steps to compensate for this psychological gap (Chen et al., 2021); on the other hand, low core self-evaluation employees have low evaluations of their own abilities, they tend to focus on their own failures and shortcomings, believe that they are not capable of achieving similar achievements as i-dealers, and are unable to change the status quo no matter how hard they try (Ng and Lucianetti, 2016b), and these feelings will lead to a negative psychological experience in comparison, thus creating a sense of relative deprivation. In contrast, individuals with high core self-evaluations tend to have a strong sense of control over their work. They believe that through hard work they will achieve similar opportunities and resources as i-dealers in the future and view i-dealers as role models. Therefore, this paper proposes that as:

*Hypothesis 1*: Perceptions of coworkers’ receiving i-deals interacting with core self-evaluations are positively related to relative deprivation. That is, under the condition of low core self-evaluations, perceptions of coworkers’ receiving i-deals are positively related to relative deprivation.

### Relative Deprivation as a Mediator

Social comparison theory indicates that unfavorable comparative information can threaten an individual’s sense of self-value and that this threat must be managed and controlled by some behavioral strategy to counteract further threats to oneself (Buunk and Gibbons, 2007).

According to Folger and Martin's (1986) model of relative deprivation response, when individuals experience feelings of deprivation, they have two options: (1) self-improvement (e.g., working harder, exhibiting more organizational citizenship behaviors) and pursuing constructive change (e.g., expressing their concerns to leaders); (2) exhibiting stress symptoms and negative attitudes (e.g., increased stress, decreased health, decreased job satisfaction counterproductive work behaviors, and workplace injuries). Employees’ positive or negative reactions to these two sorts of reactions are determined by the possibility and extent to which their situation will change in the future (Bolino and Turnley, 2009).

Duffy et al. (2002) proposed that social undermining refers to covert behaviors that chronically and intentionally impede the establishment and maintenance of positive interpersonal relationships with others, interfere with their success at work, and undermine their good reputations. As an aggressive tool, for example, withholding information, gossiping, putting others down, and “cold violence.” Social undermining not only has a negative impact on their mood, well-being, self-efficacy (Duffy et al., 2006), and interpersonal relationships (Hershcovis and Barling, 2010), but also has the potential to reduce their reputation and job performance (Duffy et al., 2006).

The sense of relative deprivation consists of two components: a cognitive component, which refers to comparisons with others and an emotional component, which refers to the negative emotions arising from perceived differences between oneself and others (Tougas et al., 2005). When employees perceptions of coworkers’ receiving i-deals are high, it means that i-deals receive more resources, opportunities, and have higher status. The horizontal comparison will generate a sense of relative deprivation. On the one hand, from the cognitive point of view, employees with low core self-evaluation believe that they are powerless to change the status quo and perceive the situation as a negative social comparison that brings individuals suspicion and competition (He et al., 2020). On the other hand, in terms of emotions, feelings of relative deprivation can bring about negative emotions of anger and resentment. Studies have shown that when employees experience a sense of relative deprivation, they will take steps to mitigate threatening feelings (Yu et al., 2018).

Therefore, individuals with low core self-evaluations believe that uncontrollable factors have deprived them of the resources and opportunities to which they are entitled, which will bring a sense of relative deprivation. The contrast effect of this upward comparison amplifies their perceptions of difference and exaggerates the threat to self-esteem and status that challenges bring (Cohen-Charash and Mueller, 2007). Because they have little potential for self-improvement through effort, they are more likely to reduce coworkers’ achievements through harm doing behaviors in order to improve their negative self-evaluations. For example, Yu et al. (2018) found that when departmental leaders faced threats to their subordinates’ self-esteem, if they did not have the potential to outperform their subordinates, they would devalue them through abusive supervision to enhance their own sense of self-worth.

Harm doing behaviors are diverse and social undermining is chosen because it is a hidden behavior and the manifestation of their adverse effects is a gradual process. In contrast to social undermining, destructive acts such as killing, physical assault, or defacing property are blatant with direct and high impacts. Social undermining does not necessarily destroy interpersonal relationships, good reputation, or working ability if committed once or twice. On the contrary, if continued over time, the adverse effect will accumulate. Social undermining as a discreet and covertly harmful behavior whose adverse effects on others are a gradual process (Reh et al., 2018). This means that the negative effects of social undermining toward core employees who have been given idiosyncratic deals will not be immediately apparent. On the one hand, the cost of social undermining does not appear in large concentrations, this reduces the guilt of the perpetrator, making it more likely to unconsciously break through the bottom line of self and continue to implement the blocking behavior, which paralyzes the perpetrator to some extent. On the other hand, it is a secure means of concealment, not easily detected. Therefore, employees with a high sense of relative deprivation are more likely to choose social undermining as a destructive behavior to hinder the success of core employees. Therefore, this paper proposes that as:

*Hypothesis 2*: Under the condition of low core self-evaluations, relative deprivation mediates the relationship between perceptions of coworkers’ receiving i-deals and social undermining.

### Conscientiousness as a Moderator

Research shows that personality characteristics play an important role in the process of social comparison (Yu et al., 2018). Conscientiousness is one of the Big Five personality factors and is related to an individual’s typical level of motivation or will, it refers to whether an individual has a high level of achievement orientation and responsible attitude toward work (Costa and McCrae, 1992).

Relative deprivation manifests through both cognitive and emotional aspects that promote social undermining, and conscientiousness can moderate the effect of relative deprivation on social undermining from both aspects. On the one hand, from the cognitive point of view, when employees have a high level of perceptions of coworkers’ receiving i-deals, they believe that they cannot change the status quo and are unable to achieve similar accomplishments as i-deals through their efforts and take social undermining behaviors to bridge the psychological gap and reduce their sense of self-threat. Existing research suggests that a person’s level of conscientiousness affects the way they work (Borghuis et al., 2017). When a sense of relative deprivation arises, conscientiousness can change employees’ cognition. Those high in conscientiousness tend to be achievement-oriented and able to work firmly toward their goals (Mount and Barrick, 1998). When faced with coworkers’ i-deals, they will persistently improve themselves to reach their goals. This will reduce their likelihood of engaging in social undermining behaviors. However, those low in conscientiousness tend to procrastinate and have less self-discipline when performing their job duties (Renn et al., 2011). The lack of self-discipline and inefficient characteristics may make it difficult for them to achieve similar accomplishments as i-dealers, which increases the likelihood of taking social undermining behaviors.

On the other hand, in terms of emotional aspect, relative deprivation contains negative emotions such as anger and resentment. When feeling a sense of relative deprivation, conscientiousness can relieve employees’ negative emotions, which will reduce social undermining behaviors. Those high in conscientiousness consider it their responsibility to do the right thing and follow ethical principles (McFerran et al., 2010) and see it as their responsibility to take care of the well-being of others and have an obligation to give back to the organization (Zhang et al., 2019). When faced with coworkers’ i-deals, this sense of responsibility and morality can alleviate the negative emotional experience of employees. At the same time, those low in conscientiousness tend to approach their work in a less disciplined manner (Moon, 2001) and they are less concerned with moral responsibility (McFerran et al., 2010). This will aggravate employees’ attention to negative emotions and engage in more social undermining behaviors. Therefore, we propose the following hypothesis:

*Hypothesis 3*: Conscientiousness moderates the relationship between relative deprivation and social undermining. When employee conscientiousness is low, the effect of relative deprivation on social undermining is stronger.

Finally, this paper proposes that under the condition of low core self-evaluations, employees have negative evaluations of themselves, perceive that they cannot reach their coworkers’ achievements no matter how hard they work so that coworkers’ i-deals can stimulate a sense of relative deprivation, a perception that they are being deprived of resources and opportunities, and trigger social undermining. Research has shown that the level of conscientiousness affects the way individuals work (Demerouti, 2006). When employees perceive that coworkers gain idiosyncratic deals, employees in low conscientiousness are more concerned with the loss of their own resources, exacerbating the sense of threat and are more likely to trigger social undermining in order to balance negative emotions. Therefore, employees in low conscientiousness who feel relatively deprived are more likely to trigger social undermining. Integrating the above four hypotheses, we further propose that as:

*Hypothesis 4*: Under the condition of low core self-evaluations, perceptions of coworkers’ receiving i-deals stimulate employees’ feelings of relative deprivation and trigger social undermining, at the same time, employees who develop feelings of relative deprivation are more likely to trigger social undermining when their conscientiousness is low.

**Figure 1:**
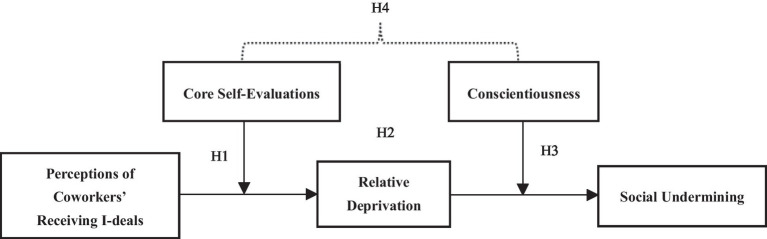
Conceptual model.

In summary, this paper proposes a two-stage moderated mediation model (see Figure 1), aiming to test whether perceived coworkers’ i-deals lead employees to engage in social undermining and to explore its mechanisms to prevent social undermining in the workplace.

## Materials and Methods

### Participants and Procedures

The study was conducted in cooperation with the Hebei Provincial Administration of Market Supervision. The sample covers six enterprises (involving financial, technology, communication, and new material industries) in Hebei province, China. Firstly, we got permission from the top management of each company for data collection. In order to ensure that the surveyed enterprises implement idiosyncratic i-deals, we randomly interviewed some employees from those enterprises. We asked managers to let employees enter a conference room in groups of 10 each, and participants sat down randomly. To ensure the validity and reliability of the data filled in by the employees, we asked the department head not to be present when the employees filled in the questionnaire. Each participant was given a souvenir with a university logo to thank them for their participation.

In order to reduce the potential common method bias, data were collected in two waves with a two-week interval (Reis and Wheeler, 1991), and questionnaires were strictly coded throughout the process. At time1, participants were asked to report their perceptions of coworkers’ receiving i-deals, core self-evaluations, relative deprivation, and demographic variables. At time 2, researchers asked them to report their conscientiousness and social undermining imposed on i-dealers. The questionnaires were collected by the surveyors on the spot after the respondents completed. The research team finally obtained 331 valid responses, with a return rate of 83%. Overall, 51.96% were male, the average age was 37.55, the average organizational tenure was 6.3 years, and 82.78% held a bachelor’s degree or above.

### Measures

In order to ensure the local applicability of the measurement, two-way translation was used (Brislin, 1980) and experts were invited to assess the appropriateness and rigor of the questionnaire. The statistical software used for data analysis was Mplus 7.4 and SPSS 23.0.

### Perceptions of Coworkers’ Receiving I-Deals

Perceptions of coworkers’ receiving i-deals were measured with Ng and Lucianetti (2016a) 6-item scale. A sample item of the perceptions of coworkers’ receiving i-deals scale reads as: “Some of my coworkers have successfully negotiated training opportunities that are different from what I have.” We used Likert-type scales ranging from 1 (strongly disagree) to 5 (strongly agree). The Cronbach’s alpha of the scale was 0.88.

### Core Self-Evaluations

Core self-evaluations were measured with Judge et al. (2003) 12-item scale. A sample item of the core self-evaluations (CSEs) scale reads as: “I am confident I get success I deserve in life.” We used a 7-point Likert scale ranging from 1 (strongly disagree) to 7 (strongly agree). The Cronbach’s alpha of the scale was 0.89.

### Relative Deprivation

Relative deprivation was measured with Callan et al. (2011) 5-item scale. A sample item of the relative deprivation scale reads as: “I feel deprived when I think about what I have compared to what other people like me have.” We used a 6-point Likert scale ranging from 1 (strongly disagree) to 6 (strongly agree). The Cronbach’s alpha of the scale was 0.89.

### Conscientiousness

Conscientiousness was measured with the 8-item scale by Saucier (1994) from the Big Five personality measurement by Goldberg (1992). Respondents rated the accuracy of keywords describing their characteristics, including “practical,” “systematic,” and “efficient.” Responses to these items were made on 7-point scales 1 (extremely inaccurate) and 7 (extremely accurate). The Cronbach’s alpha of the scale was 0.88.

### Social Undermining

Social undermining was measured with Duffy et al. (2012) 6-item scale. The introductory sentence is “To what extent do you engage in the following behaviors to i-dealers in your organization?” A sample item of the social undermining scale reads as: “I sometimes talk bad about them behind their backs.” The items had seven Likert-type response options. The Cronbach’s alpha of the scale was 0.89.

### Control Variables

This study controlled for the effects of employee demographics, including gender, age, education, and organizational tenure. In addition, we add dummy variables of enterprises to rule out the influence of situational variables at the organizational level.

## Results

### Confirmatory Factor Analysis

First, this study conducted an analysis (CFA) to examine the validity of the five key variables in the model (perceptions of coworkers’ receiving i-deals, relative deprivation, social undermining, core self-evaluations, and conscientiousness). We used Chi-square, the comparative fit index (CFI), the root mean square error of approximation (RMSEA), and the standardized root mean residual (SRMR) to assess model fit. As shown in Table 1, the results indicated that the absolute and relative fit indices of the five-factor model (*χ*^2^ = 1245.940, *df* = 619, *CFI* = 0.900, *RMSEA* = 0.055, *IFI* = 0.901) were closer to the standard values compared to the four competing models. Therefore, the scale used in this study has good discriminant validity. The CMV factor was subsequently added to the five-factor model to assess the common method bias. It was found that the six-factor model had limited improvement in *RMSEA*, *CFI*, and *TLI*, all of which were less than 0.05 (Bagozzi and Yi, 1990), indicating that there was no serious common method bias in this study.

**Table 1:** Results of confirmatory factor analyses.

Model	*χ* ^2^	*df*	*χ*^2^/*df*	*RMSEA*	*CFI*	*IFI*	*TLI*
One-factor model	5405.043	629	8.593	0.152	0.240	0.244	0.195
Two-factor model	4369.751	628	6.958	0.134	0.404	0.408	0.368
Three-factor model	3358.578	626	5.365	0.115	0.565	0.568	0.537
Four-factor model	2387.548	623	3.832	0.093	0.719	0.721	0.700
Five-factor model	1245.940	619	2.013	0.055	0.900	0.901	0.893
Six-factor model	957.523	584	1.640	0.044	0.941	0.941	0.932

One-factor model = PCRI + CSEs + RD + C + SU; two-factor model = PCRI + CSEs + RD + C, and SU; three-factor model = PCRI; CSEs + RD + C, and SU; four-factor model = PCRI, CSEs + C, RD, and SU; five-factor model = PCRI, CSEs, RD, C, and SU; PCRI, perceptions of coworkers’ receiving i-deals; CSEs, core self-evaluations; RD, relative deprivation; C, conscientiousness; and SU, social undermining.

### Descriptive Analyses

Table 2 shows means, standard deviations, and correlation analyses. Perceptions of coworkers’ receiving i-deals are positively correlated with relative deprivation (*r* = 0.385, *p* < 0.001) and social undermining (*r* = 0.223, *p* < 0.01). Relative deprivation is positively correlated with social undermining turn (*r* = 0.318, *p* < 0.001). These results preliminary provided support for subsequent hypotheses testing.

**Table 2:** Means, SDs, and correlation analyses.

Variable	*Mean*	*SD*	1	2	3	4	5	6	7	8	9	10
1. Gender	1.52	0.50	-									
2. Age	2.76	1.21	0.040	-								
3. Education	3.25	0.93	0.084	−0.144^**^	-							
4. Tenure	2.82	1.28	0.038	0.353^***^	−0.060	-						
5. Type of enterprises	2.54	1.11	−0.090	−0.004	−0.152^*^	0.065	-					
6. Perceptions of coworkers’ receiving i-deals	3.62	0.84	0.063	−0.003	−0.081	0.001	0.364^*^	-				
7. Relative deprivation	3.97	1.32	0.001	−0.140^*^	−0.043	−0.068	0.385^*^	0.385^***^	-			
8. Core self-evaluations	3.76	1.30	0.050	0.085	0.017	−0.001	−0.057	−0.057	−0.025	-		
9. Social undermining	4.04	1.57	0.116^*^	−0.074	0.010	−0.072	0.151^*^	0.223^***^	0.318^***^	0.129^*^	-	
10. Conscientiousness	3.88	1.24	−0.082	0.076	−0.011	−0.043	−0.082	−0.082	−0.074	−0.033	−0.153^**^	-

I-deals, idiosyncratic deals; SD, standard deviation; and *N* = 331. Two tailed.

* *p* < 0.05;

** *p* < 0.01;

*** *p* < 0.001.

### Test of Hypotheses

We use Mplus7.4 software and apply Bootstrap sampling interval estimation method for hypothesis testing, setting the number of replicate samples at 20000, and if 95% confidence interval (CI) does not include 0, the indirect effect is significant.

**Figure 2:**
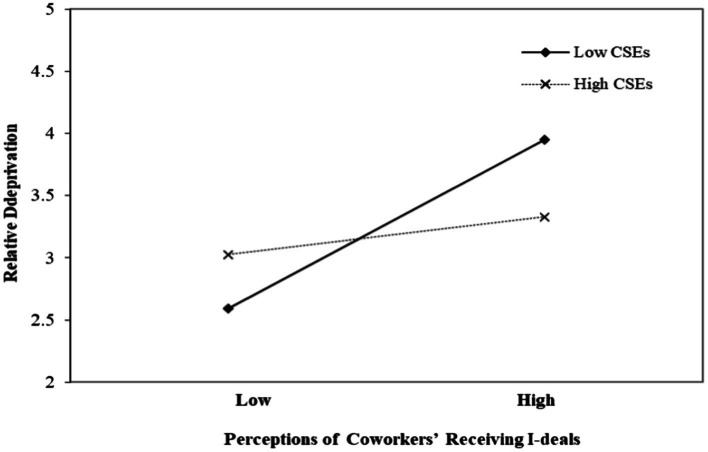
The moderating effect of core self-evaluations (CSEs) on the relationship between perceptions of coworkers’ receiving i-deals and relative deprivation.

In the first step, we tested H1, X*W1 → M. The results in Table 3 showed that the interaction of perceptions of coworkers’ receiving i-deals (X) and core self-evaluations (W1) is negatively related to relative deprivation (M; *β* = −0.265, *p* < 0.001). Figure 2 shows the plot of the effect of perceptions of coworkers’ receiving i-deals (X) on relative deprivation (M) for the two conditions of low and high core self-evaluations (W1). As shown in Figure 2, the effect was significant and positive only under the condition of low core self-evaluations. Hence, H1 was supported.

**Table 3:** Bootstrapping results for moderating effect of W1 and W2.

Path	Effect	Boot S.E.	[95% CI]
X → M	0.415^***^	0.081	[0.249, 0.567]
X * W1 → M	−0.265^***^	0.059	[−0.378, −0.144]
M → Y	0.254^**^	0.083	[0.088, 0.415]
M * W2 → Y	−0.142^*^	0.062	[−0.257, −0.014]

*N* = 331. Two tailed; Bootstrap = 20,000.

* *p* < 0.05;

** *p* < 0.01;

*** *p* < 0.001.

In the second step, we tested H2, X*W1 → M → Y. Table 4 shows that when the core self-evaluations (W1) are low, the indirect effect is significant (P_YM_*P_MX_ = 0.757 × 0.326 = 0.247), 95% CI = [0.119, 0.416]. This implies that under the condition of low core self-evaluations (W1), the high perceptions of coworkers’ receiving i-deals (X) will trigger social undermining (Y) by generating a stronger sense of relative deprivation (M). Therefore, H2 is supported.

**Table 4:** Analysis results of moderated mediation effect (core self-evaluations).

Variable		Stage 1	Stage 2	Direct effects	Indirect effects	Total effects
		X → M	M → Y	X → Y	(P_YM_^*^P_MX_)	(P_YX_+[P_YM_^*^P_MX_])
		[95% CI]	[95% CI]	[95% CI]	[95% CI]	[95% CI]
Core self-evaluations	High	0.072	0.326^***^	0.246^*^	0.024	0.269^*^
		[−0.136, 0.280]	[0.167, 0.479]	[0.030, 0.447]	[−0.040, 0.105]	[0.049, 0.471]
	Low	0.757^***^	0.326^***^	0.246^*^	**0.247** ^**^	0.493^***^
		[0.527, 0.980]	[0.167, 0.479]	[0.030, 0.447]	**[0.119, 0.416]**	[0.291, 0.691]
	Difference	−0.685^***^	0	0	**−0.223** ^**^	−0.223^**^
		[−0.978, −0.373]	-	-	**[−0.408, −0.099]**	[−0.408, −0.099]

*N* = 331. Two tailed; Bootstrap = 20,000.

* *p* < 0.05;

** *p* < 0.01;

*** *p* < 0.001.

Bold values prove the hypothesis 1.

**Figure 3:**
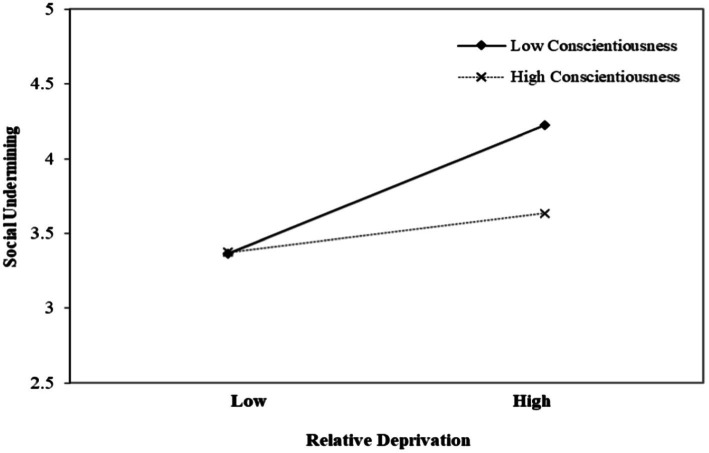
The moderating effect of conscientiousness on the relationship between relative deprivation and social undermining.

In the third step, we tested H3, the moderating effect of conscientiousness (W2). The result in Table 5 revealed that the relationship between relative deprivation (M) and social undermining (Y) was significant and positive when conscientiousness (W2) was low (*β* = 0.436, *p* < 0.001) but was not significant when conscientiousness (W2) was high (*β* = 0.037, n.s.). Figure 3 shows a plot of this relationship. Hence, H3 was supported.

**Table 5:** Analysis results of moderated mediation effect (conscientiousness).

Variable		Stage 1	Stage 2	Direct effects	Indirect effects	Total effects
		X → M	M → Y	X → Y	(P_YM_^*^P_MX_)	(P_YX_+[P_YM_^*^P_MX_])
		[95% CI]	[95% CI]	[95% CI]	[95% CI]	[95% CI]
Conscientiousness	High	0.380^***^	0.037	0.226^*^	0.014	0.240^*^
		[0.443, 0.756]	[−0.231, 0.285]	[0.029, 0.411]	[−0.138, 0.180]	[0.033, 0.445]
	Low		0.436^***^		**0.166** ^**^	0.391^***^
			[0.258, 0.606]		**[0.147, 0.405]**	[0.295, 0.668]
	Difference	0	−0.399^**^	0	**−0.152** ^*^	−0.152^*^
		-	[−0.672, −0.090]	-	**[−0.435, −0.060]**	[−0.435, −0.060]

*N* = 331. Two tailed; Bootstrap = 20,000.

* *p* < 0.05;

** *p* < 0.01;

*** *p* < 0.001.

Bold values prove the hypothesis 2.

**Figure 4:**
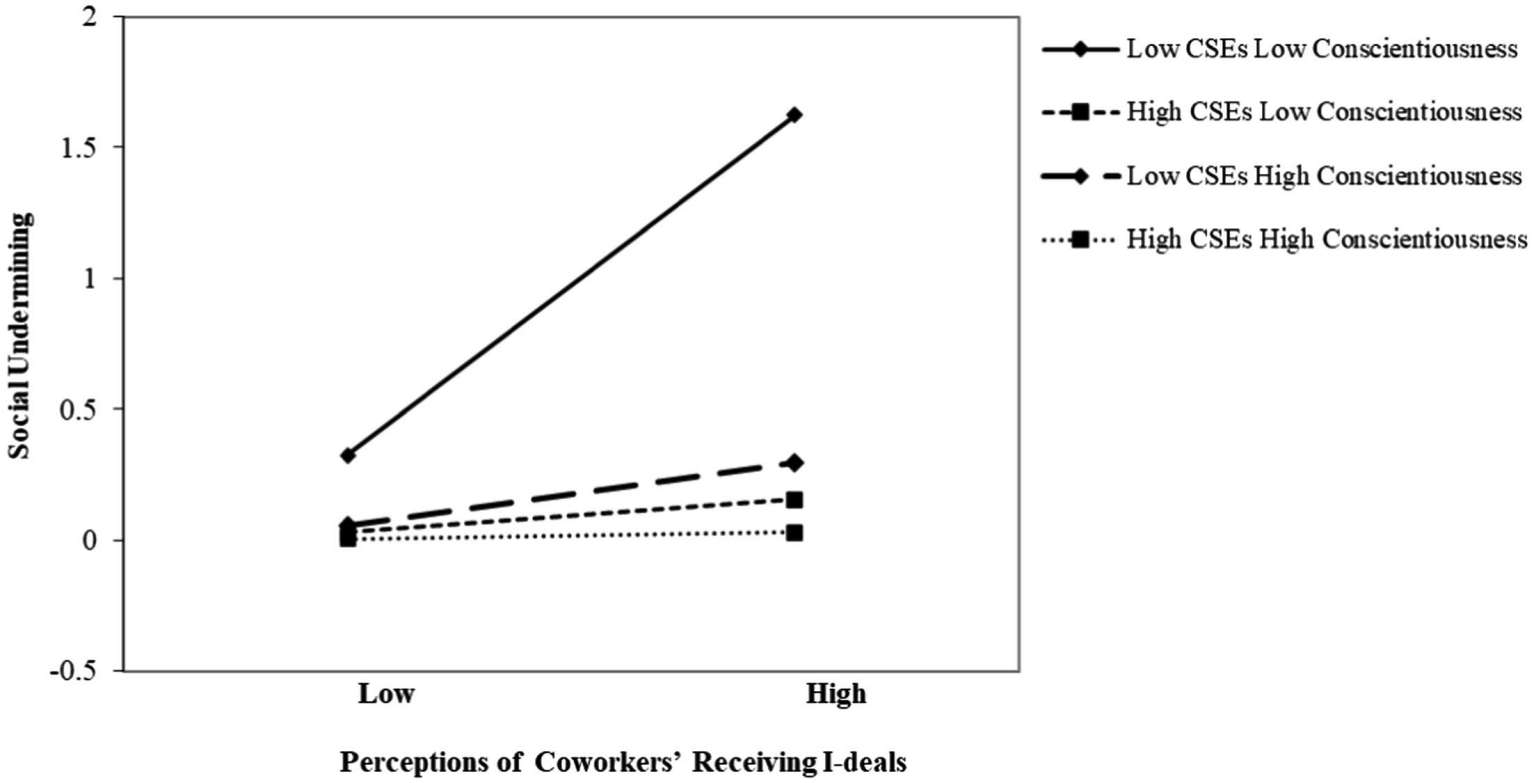
Moderated indirect effect of perceptions of coworkers’ receiving i-deals on social undermining (*via* relative deprivation) at low and high levels of CSEs and conscientiousness.

In the fourth step, we tested the indirect effect hypothesis 4 of the full model, X*W1 → M*W2 → Y. As shown in Table 6, the indirect effect of perceptions of coworkers’ receiving i-deals (X) on social undermining (Y) through relative deprivation (M) was significant (P_YM_*P_MX_ = 0.757 × 0.429 = 0.325, 95% CI = [0.175, 0.526], and the total effect is 0.572, 95% CI = [0.349, 0.798]). Figure 4 shows the plot of the indirect effect of perceptions of coworkers’ receiving i-deals (X) on social undermining (Y) through relative deprivation (M) for the four combinations of low and high core self-evaluations (W1) and conscientiousness (W2). As shown in Figure 4, the indirect effect was significant and positive only under the combination of low core self-evaluations (W1) and low conscientiousness (W2); under the other conditions, there was no significant indirect effect of perceptions of coworkers’ receiving i-deals (X) on social undermining (Y). Hence, H4 was supported.

**Table 6:** Analysis results of moderated mediation effect of two stage.

Variable		Stage 1	Stage 2	Direct effects	Indirect effects	Total effects
		X → M	M → Y	X → Y	(PYM^*^PMX)	(PYX+[PYM^*^PMX])
		[95% CI]	[95% CI]	[95% CI]	[95% CI]	[95% CI]
High core self-evaluations	High conscientiousness	0.072	0.078	0.247^*^	0.006	0.252^*^
		[−0.136, 0.280]	[−0.185, 0.332]	[0.042, 0.446]	[−0.015, 0.077]	[0.051, 0.448]
	Low conscientiousness	0.072	0.429^***^	0.247^*^	0.031	0.278^*^
		[−0.136, 0.280]	[0.248, 0.600]	[0.042, 0.446]	[−0.054, 0.135]	[0.061, 0.483]
Low core self-evaluations	High conscientiousness	0.757^***^	0.078	0.247^*^	0.059	0.306^*^
		[0.527, 0.980]	[−0.185, 0.332]	[0.042, 0.446]	[−0.136, 0.268]	[0.064, 0.549]
	Low conscientiousness	0.757^***^	0.429^***^	0.247^*^	**0.325** ^***^	**0.572** ^***^
		[0.527, 0.980]	[0.248, 0.600]	[0.042, 0.446]	**[0.175, 0.526]**	**[0.349, 0.798]**

*N* = 331. Two tailed; Bootstrap = 20,000.

* *p* < 0.05;

*** *p* < 0.001.

Bold values prove the hypothesis 4.

## Discussion

Drawing on social comparison theory, this study proposed and tested a moderated mediation model to explain the mechanisms through which perceptions of coworkers’ receiving idiosyncratic deals affect social undermining and how to resolve this negative impact. The results of the study found that under the condition of low core self-evaluations, perceptions of coworkers’ receiving idiosyncratic deals positively influenced relative deprivation and promoted social undermining. At the same time, conscientiousness negatively moderated the relationship between feelings of relative deprivation and social undermining. The study reveals the negative consequences of idiosyncratic deals from a third-party perspective, providing theoretical and practical insights into the study of idiosyncratic deals and social undermining.

### Theoretical Implications

This paper has several theoretical implications. First, it reveals the incentive dilemma of idiosyncratic deals within the organization. Due to limited resources are often invested in a few core employees in order to maximize organizational benefits. This practice certainly has good effects on the organization: it not only creates an atmosphere of striving for excellence within the organization, on the other hand, but also makes i-dealers representative of the organization’s goals, providing a model for other employees. However, we found that i-dealers who receive idiosyncratic deals are more likely to be treated as objects of social undermining. As Lam et al. (2011) suggest, core employees are not as glamorous as they appear, and organizations should pay special attention to their victimized behaviors. Moreover, this negative behavior then affects the organizational climate, so that i-dealers are forced to deal with interpersonal distractions. At the same time, in a highly collaborative organization, if other colleagues do not cooperate, i-dealers will not be able to maintain high performance and high work motivation (He et al., 2021). This will affect organizational performance and put it in a core employee motivation dilemma.

Second, this study contributes to i-deals literature by exploring the negative effect of coworker idiosyncratic deals on non-recipients during interpersonal interactions. Since satisfying the individualized needs of core employees to achieve a win-win situation for both the organization and the idiosyncratic dealers, existing research has focused on the positive effects of idiosyncratic deals on recipients, relatively neglecting the feelings of third-party employees in organizational tripartite contexts. Only a few studies found that idiosyncratic deals can reduce peer motivation (Giarratana et al., 2018), deviant behaviors (Kong et al., 2020), and performance (Abdulsalam et al., 2021). However, these studies ignore its potential negative effects during interpersonal interactions. In response to the call of Ng (2017) for more research to explore the potential negative effects of idiosyncratic deals, this study finds that idiosyncratic deals will also lead to social undermining, enriching the expansion of research on idiosyncratic deals’ negative effects of interpersonal relationships.

Third, this study contributes to social comparison theory by revealing that core self-evaluations and relative deprivation are important mechanisms that promote social undermining for coworkers’ idiosyncratic deals. The findings show that under the condition of low core self-evaluations, perceptions of coworkers’ receiving idiosyncratic deals positively affect the sense of relative deprivation and generates social undermining. Although some scholars have explored the negative effects of idiosyncratic deals (Ng, 2017; Abdulsalam et al., 2021; Zhang et al., 2021), there remains a critical question that needs to be addressed as: what are the negative effects of coworker idiosyncratic deals trading during interpersonal interactions and under what boundary conditions do perceptions of coworkers’ receiving idiosyncratic deals have negative effects? According to social comparison theory, personality characteristics play an important role in the process of social comparison. We found that this depends on employees’ core self-evaluations. For individuals with low core self-evaluations, the presence of i-dealers can be a threat to their workplace status. In other words, individuals’ core self-evaluations are important boundary conditions for the negative effects of idiosyncratic deals. This finding strongly supports the view of Collins (1996) that self-evaluations can have a decisive impact on the outcome of upward social comparison. The study extends the application of social comparison theory in the field of idiosyncratic deal research.

Fourth, personality characteristics play an important role in the process of attribution (Yu et al., 2018), this paper reveals that conscientiousness moderators the effect between relative deprivation and social undermining. Specifically, we showed that those high in conscientiousness tend to be confident in fact of challenges and work harder. In contrast, employees in low conscientiousness are considered to be unorganized and undisciplined, those developed feeling of relative deprivation in these characteristics are more likely to engage in social undermining. In this way, we demonstrated that not all employees will engage in social undermining when they perceive coworkers’ idiosyncratic deals.

### Practical Implications

The results of this study also provide some practical insights for organizations and employees. When organizations need to use idiosyncratic deals to motivate and attract core employees, the following approaches can be taken to minimize negative feedback from non-recipients.

First, as an important psychological resource for individuals, core self-evaluations can alleviate the generation of relative deprivation. Therefore, although core self-evaluations have individual stability and innate nature, external factors also have an important influence on it. Leaders can improve employees’ core self-evaluations by shaping good leadership styles. For example, benevolent leaders can effectively stimulate employees’ self-esteem and self-efficacy, thus increasing their overall core self-evaluations levels, while abusive leaders tend to undermine employees’ self-confidence, making their core self-evaluations lower (Antonakis et al., 2012).

Second, managers can create an open access to idiosyncratic deals, instead of filling the psychological gap through social undermining. On the one hand, enterprises can set up unions to collect employees’ voice, communicate with management to help managers choose more effective work arrangements and personnel policies (Fang et al., 2019), and improve the motivational effect of idiosyncratic deals. On the other hand, the existing incentive system only focuses on the core employees. Therefore, managers can expand the range of recipients of idiosyncratic deals and set different assessment standards according to the difference of employees’ ability and then reward them according to the elite incentive method, so that employees in any echelon can get idiosyncratic deals, thus activating the sense of alignment and excellence within the organization.

Third, employees who have obtained idiosyncratic deals should increase extra-role behaviors (e.g., organizational citizenship behaviors). Because the i-dealers are seen as having more rewards by the non-recipients, increasing payoffs can not only indirectly restore the “give-reward” imbalance of the non-recipients, but also help the i-dealers establish good collegial relationships and reduce resistance at work.

In addition, strict rules and regulations can be established and employees can be informed of the cost of social undermining to reduce the destructive behavior among employees; regular internal seminars are held, where i-dealers take the initiative to share their experience and skills, so that employees can feel that the performance gap is narrowing; and actively create a relaxed and harmonious team atmosphere, and hold regular group activities to increase the intimacy of the relationship between employees.

### Limitations and Future Research

There are some limitations in this study. First, this study explored the relationship between coworker idiosyncratic deals and social undermining based on social comparison theory. Research shows that different types of idiosyncratic deals can have different motivational effects on employees. Future research could explore in depth the effects of different types of idiosyncratic deals, such as the impact of flexible idiosyncratic deals and developmental idiosyncratic deals, pre-work (ex-ante) idiosyncratic deals, and post-work (ex-post) idiosyncratic deals on for non-recipients.

Second, this study considers the boundary conditions and the mechanisms underlying the social undermining of perception coworkers’ receiving idiosyncratic deals from the perspective of personality characteristics. Although from the perspective of social comparison theory, personality characteristics are a non-negligible factor in the process of individual social comparison. However, compared with the situational variables at the organizational level, such as organizational climate, organizational mechanism design, and organizational context, personality characteristics have strong immutability, and the boundary role of organizational mechanism, organizational climate, and organizational context in the process of idiosyncratic deals generating social undermining can be further explored in the future to expand the practical value and theoretical significance of idiosyncratic deals.

Third, although this study adopted a multi-temporal data collection approach, the causal relationship between the variables is difficult to be fully determined due to the limitations of the questionnaire paradigm. It is recommended that future studies use a combination of experiments (e.g., showing subjects videos of their colleagues negotiating idiosyncratic deals with their leaders and successfully reaching consensus) and questionnaires to further explore this issue in order to improve the credibility of the findings.

Finally, our study sample is limited to the Chinese context, so it is uncertain to what extent these findings can be generalized beyond the Chinese culture, and the generalizability of the findings in the Western context needs to be further verified. Future cross-cultural studies can be conducted to better explore the mechanisms and boundary conditions of the impact of idiosyncratic deals on social undermining in different cultural contexts.

## Conclusion

Drawing on social comparison theory, this study revealed the mechanisms of the mediating effects of the relative deprivation between perceptions of coworkers’ receiving idiosyncratic deals and social undermining. This study also found that core self-evaluations and conscientiousness play moderating roles. The results advance understandings of how perceptions of coworkers’ receiving idiosyncratic deals impact social undermining, providing theoretical and practical insights for human resource personnel.

## Data Availability Statement

The raw data supporting the conclusions of this article will be made available by the authors, without undue reservation.

## Ethics Statement

Written informed consent was obtained from the individual(s) for the publication of any potentially identifiable images or data included in this article.

## Author Contributions

All authors listed have made a substantial, direct and intellectual contribution to the work, and approved it for publication.

## FUNDING

This work was supported by the National Natural Science Foundation of China (71872111) and Humanities and Social Sciences Foundation of Ministry of Education of China (16YJA630036).

## Conflict of Interest

The authors declare that the research was conducted in the absence of any commercial or financial relationships that could be construed as a potential conflict of interest.

## Publisher’s Note

All claims expressed in this article are solely those of the authors and do not necessarily represent those of their affiliated organizations, or those of the publisher, the editors and the reviewers. Any product that may be evaluated in this article, or claim that may be made by its manufacturer, is not guaranteed or endorsed by the publisher.
